# Predictors associated with critical care need and in-hospital mortality among children with laboratory-confirmed COVID-19 infection in a high HIV infection burden region

**DOI:** 10.3389/fped.2023.1252886

**Published:** 2023-09-07

**Authors:** Liliane N. Byamungu, Jean B. Nachega, Ashendri Pillay, Patrick D. M. C. Katoto, Prakash Jeena, Lindsay Zurba, Refiloe Masekela

**Affiliations:** ^1^ Department of Pediatrics and Child Health, School of Clinical Medicine, College of Health Sciences, University of KwaZulu Natal, Durban, South Africa; ^2^ Department of Paediatrics, Department of Medicine, Catholic University of Bukavu, Bukavu, Democratic Republic of Congo; ^3^ Department of Epidemiology, Infectious Diseases and Microbiology, Center for Global Health, University of Pittsburgh, Pittsburgh, PA, United States; ^4^ Department of Medicine, Faculty of Medicine and Health Sciences, Stellenbosch University, Cape Town, South Africa; ^5^ Department of Epidemiology, Johns Hopkins Bloomberg School of Public Health, Baltimore, MD, United States; ^6^ Department of International Health, Johns Hopkins Bloomberg School of Public Health, Baltimore, MD, United States; ^7^ Cochrane South Africa, South African Medical Research Council, Cape Town, South Africa; ^8^ Department of Global Health, Faculty of Medicine and Health Sciences, Stellenbosch University, Cape Town, South Africa; ^9^ Centre for Tropical Diseases and Global Health and Faculty of Medicine, Catholic University of Bukavu, Bukavu, Democratic Republic of Congo; ^10^ Department of Paediatrics and Child Health, University of Kwazulu-Natal, Education for Health Africa, Durban, South Africa

**Keywords:** SARS-CoV-2, intensive care, comorbidity, death, sub-Saharan Africa

## Abstract

**Introduction:**

Despite the extra mortality associated with COVID-19 death globally, there is scant data on COVID-19-related paediatric mortality in Sub-Saharan Africa. We assessed predictors of critical care needs and hospital mortality in South African children with laboratory-confirmed SARS-CoV-2 infection in region with high HIV infection burden.

**Methods:**

We conducted a secondary multicentre analysis of the AFREhealth cohort (a multinational, multicentre cohort of paediatric COVID-19 clinical outcomes across six African countries) of children admitted to the Inkosi Albert Luthuli, a quaternary hospital in KwaZulu-Natal, South Africa, with confirmed RT-PCR between March 2020 and December 2020. We constructed multivariable logistic regression to explore factors associated with the need for critical care (high care/ intensive care hospitalisation or oxygen requirement) and cox-proportional hazards models to further assess factors independently associated with in-hospital death.

**Results:**

Of the 82 children with PCR-confirmed SARS-CoV-2 infection (mean ± SD age: 4.2 ± 4.4 years), 35(42.7%) were younger than one year, 52(63%) were female and 59(71%) had a pre-existing medical condition. Thirty-seven (45.2%) children required critical care (median (IQR) duration: 7.5 (0.5–13.5) days) and 14(17%) died. Independent factors associated with need for critical care were being younger than 1 year (aPR: 3.02, 95%CI: 1.05–8.66; *p* = 0.04), having more than one comorbidity (aPR: 2.47, 95%CI: 1.32–4.61; *p* = 0.004), seizure (aPR: 2.39, 95%CI: 1.56–3.68; *p* < 0.001) and impaired renal function. Additionally, independent predictors of in-hospital mortality were exposure to HIV infection (aHR: 6.8, 95%CI:1.54–31.71; *p* = 0.01), requiring invasive ventilation (aHR: 3.59, 95%CI: 1.01–12.16, *p* = 0.048) and increase blood urea nitrogen (aHR: 1.06, 95%CI: 1.01–1.11; *p* = 0.017). However, children were less likely to die from COVID-19 if they were primarily admitted to quaternary unit (aHR: 0.23, 95%CI: 0.1–0.86, *p* = 0.029).

**Conclusion:**

We found a relatively high hospital death rate among children with confirmed COVID-19. During COVID-19 waves, a timely referral system and rapid identification of children at risk for critical care needs and death, such as those less than one year and those with comorbidities, could minimize excess mortality, particularly in high HIV-infection burden countries.

## Introduction

The devastating impact of the COVID-19 pandemic has resulted in a staggering death toll of 6.9 million as of May 2023, according to the World Health Organization (WHO) (1). Alarming statistics reveal that the virus has claimed the lives of 17,400 individuals under the age of 20. However, these numbers only account for the direct impact of the virus, with the indirect effects garnering less attention. Current evidence supports that middle-income countries have been disproportionately affected by the indirect impact, with 81% of the 14.9 million excess deaths occurring in these regions between 2020 and 2021 (2). The impact of COVID-19 on healthcare systems has been significant, with economic implications that have hit low-income settings the hardest, where patients with additional comorbidities and limited access to appropriate care are most vulnerable (3, 4).

Moreover, despite the easing of lockdown measures, the emergence of novel variants of concern (VOCs) means that SARS-CoV-2 remains a global threat (3, 5, 6). However, previous data have shown limitations (direct and indirect impact) in generalising the evidence to under-represented African settings and other low to middle income countries, where healthcare limitations are already in play (7). A bibliometric analysis of COVID-19 research in Africa revealed that only 20.5% of studies included the African continent, with most of the research focusing on “country preparedness and response” (24.9%) and “the direct and indirect health impacts of the pandemic” (21.6%). Regrettably, very few studies focused on both the direct and indirect impacts on the paediatric population (8). The lack of primary research on children and adolescents who have been victims of varied experiences such as social isolation and who are at the intersection of a number of markers that cause inequity and asymmetry in health/disease interactions highlights a significant place in the generation of empirical evidence (9).

In our parent study, we explored clinical manifestations, outcomes, and factors associated with outcomes among children and adolescents hospitalized with COVID-19 in six countries in sub-Saharan Africa (The AFREhealth Cohort) (10). Our hypothesis was that the presence of any comorbidity and very limited access to high-quality paediatric intensive care could potentially affect the clinical course of SARS-CoV-2 in children living in Sub-Saharan Africa. We found that the morbidity and mortality rates among hospitalized children and adolescents with COVID-19 were substantially higher than those reported in non-African settings (11), and were associated with age younger than 1 year and selected non-communicable disease comorbidities (chronic kidney diseases, chronic lung diseases, haematological disorders, liver diseases and chronic neurologic diseases).

Our study postulated that children living with or exposed to HIV infection in regions with a significant HIV burden would face a higher risk of severe COVID-19 outcomes. Several potential explanations support this heightened risk (12–14), necessitating further investigation into outcomes for HIV-exposed children. Understanding determinants of severe COVID-19 outcomes in these resource-constrained settings could inform preventive strategies like vaccination and mitigate healthcare delays through enhanced referral systems and triage algorithms. Hence, we aimed to delve into factors tied to critical care needs and in-hospital mortality among children with confirmed SARS-CoV-2 infection in KwaZulu-Natal, South Africa, a region with a high HIV infection burden.

## Methods

### Settings, participants and study design

Detailed information about the AFREhealth Cohort (including participating health care facilities characteristics such as names, locations, urban vs. rural settings, and public vs. private status) is available in the published parent study (10). Briefly, this retrospective record review was a multi-country cohort study that included hospitalized children and adolescents between the ages of 0 and 19 with confirmed SARS-CoV-2 infection through reverse transcriptase polymerase chain reaction (PCR) testing. The study was approved by institutional and/or national research ethics committees and/or regulatory bodies in participating countries, and followed the Strengthening the Reporting of Observational Studies in Epidemiology (STROBE) reporting guideline (BREC/00002196/2020).

This study included all children and adolescents with confirmed SARS-CoV-2 infection who were admitted (directly or through transfer from a regional facility) to a quaternary facility in the province of Kwazulu-Natal, Inkosi Albert Luthuli Central Hospital (IALCH) between March 1, 2020 and December 31, 2020. IALCH receives referrals from all regional and tertiary hospitals in the province (Greys Hospital and its regional hospitals, Mahatma Gandhi Hospital, Prince Mshiyeni Hospital, RK Khan hospital, King Edward VIII Hospital, Addington Hospital). Data on race and ethnicity were not collected because the racial profile across was more than 78% Black or African descent, and the ethnic diversity across was too broad (South African and foreign ethnic groups combined) for meaningful categorization or analysis.

### Independent and dependent variables

Independent variables included demographic and clinical data such as age, sex, pre-existing comorbidities, COVID-19 severity stage at admission, diagnosis of multisystem inflammatory syndrome in children (MIS-C) (1), and imaging, biological and microbiological assessments. They were extracted from national or institutional COVID-19 data sets and/or hospital records using WHO paediatric COVID-19 case report forms. Cases were characterized as suspected MIS-C when at least two required multisystem abnormalities were documented in the medical records and/or databases from which study data were extracted, in addition to fulfilling WHO criteria for MIS-C diagnosis and confirmation of COVID-19 through positive PCR. For a diagnosis of MISC in individuals aged 0–19 years, the patient must have a fever lasting more than three days and at least two of the following symptoms: rash or bilateral non-purulent conjunctivitis or muco-cutaneous inflammation signs (oral, hands or feet), hypotension or shock, features of myocardial dysfunction, pericarditis, valvulitis, or coronary abnormalities (including Echocardiogram/echo findings or elevated Troponin/NT-proBNP), evidence of coagulopathy (by Prothrombin Time /PT, Partial Thromboplastin Time /PTT, elevated d-Dimers), acute gastrointestinal problems (diarrhoea, vomiting, or abdominal pain), and elevated markers of inflammation such as Erythrocyte Sedimentation Rate/ESR, C-reactive protein, or procalcitonin. In addition, there should be no other obvious microbial cause of inflammation, including bacterial sepsis, staphylococcal or streptococcal shock syndromes, and evidence of COVID-19 (RT-PCR, antigen test, or serology positive), or likely contact with patients with COVID-19.

Children were classified as HIV-exposed if their mother had a confirmed HIV infection during pregnancy or delivery or breastfeeding. Those who tested positive on an HIV PCR test were classified as living with HIV infection (HIV positive). The study's dependent variables comprised of the requirement for critical care, such as hospitalization with the need for oxygen supplementation, admission to the intensive care unit (ICU), invasive mechanical ventilation, and/or in-hospital mortality. Additionally, the length of hospital stay was measured as a secondary outcome.

### Statistical analysis

We analysed the data using various statistical methods, including reporting frequencies and percentages for categorical variables, means and standard deviations (SDs), or medians and interquartile ranges (IQRs) for continuous variables as appropriated. To test for associations, we used chi-square (exact), t-tests, and Wilcoxon rank sum tests, where applicable. To identify independent predictors associated with the odds for the need for critical care and the hazard of death among PCR-confirmed children hospitalized at the quaternary hospital, we constructed multivariable regressions models. Considering that only a very number of selected patients might be admitted at a quaternary health care facility, different models exploring different factors associated with COVID-19 severity and susceptible of impacting on the clinical management such as sociodemographic characteristics, clinical presentation, history of comorbidity, biological markers, microbiology and imaging findings as well as type of treatment received were preferred to preserve the stability of models as well as the overall study power. The final models contained age (as continuous), sex, and other predictors of *a priori* clinical or epidemiological relevance. We represented the strength of the relationship as adjusted prevalence ratios (aPR) for modified Poisson regression (Poisson regression with log link and robust sandwich standard error) or adjusted hazard ratios (aHR) for Cox-proportional regression and corresponding 95% confidence intervals (CI). The utilization of Robust Poisson was deemed necessary due to convergence difficulties encountered with the binomial model utilizing the log link (log-binomial) (15, 16). We used Stata software version 14.1 (Stata, College Station, TX) for all data analysis, and reported *p*-values are exact and two-tailed, with values < 0.05 considered statistically significant. GraphPad Prism V 9.0 was used for visual representation.

The binomial model with the log link has convergence issues with many covariates. Therefore, the adjusted RR (ARR) or adjusted PR (APR) can be challenging to obtain in some situations. The modified Poisson regression (Poisson regression with log link and robust sandwich standard error) can be used as an alternative.

## Results

### Sociodemographic, clinical and radiologic characteristics of PCR-confirmed SARS-CoV-2 children by survival status

The study analysed data from 82 children aged 12 years and younger who were admitted directly from home to IALCH (57.3%) or transferred from tertiary and secondary referral facilities (42.7%). The age range of the cohort was 3 months to 12 years, with a median age of 4.19 years (SD 4.38 years) (Table 1). The majority of the cohort was female (52 females vs. 30 males) and there was a significant likelihood of death in females (13 vs. 1, *p* = 0.027). Most of the children (55%) required low care management in general wards, while 19/82 children required ICU admission, which was significantly associated with mortality (23%). One-third of the children had radiological signs of pneumonia, while 7/82 (8%) had clinical and laboratory findings suggestive of MISC. The overall mortality rate was significantly high at 17%. Symptoms at presentation associated with death were respiratory distress, seizures, floppiness/hypotonia, and hypotension. Children who presented with fever had a higher risk of death (30%), but this was not statistically significant. Half of the children had a comorbidity, with 17/50 (34%) having two or more comorbidities. One in five (22%) children had reported exposure to HIV infection and one-third of them died (33%). Three children tested positive for HIV infection, and one of them died.

**Table 1:** Sociodemographic and clinical characteristics of PCR-confirmed SARS-CoV-2 children hospitalised at a quaternary health care facility in KwaZulu-Natal South Africa during the first and second waves of COVID-19 pandemic and classified by survival status.

Variables	All	Alive	Dead	*p*-values
	*N* = 82	*N* = 68	*N* = 14	
Primary admission at IALCH/yes	47 (57.3%)	41 (60.3%)	6 (42.9%)	0.37
Age (years)	4.19 (4.38)	4.28 (4.39)	3.71 (4.47)	0.67
Age group				0.63
<1 yearr.	35 (42.7%)	27 (39.7%)	8 (57.1%)	
1–4 years.	15 (18.3%)	14 (20.6%)	1 (7.14%)	
5–9 years	15 (18.3%)	13 (19.1%)	2 (14.3%)	
10–12 years.	17 (20.7%)	14 (20.6%)	3 (21.4%)	
Gender				**0** **.** **027**
Female	52 (63.4%)	39 (57.4%)	13 (92.9%)	
Male	30 (36.6%)	29 (42.6%)	1 (7.14%)	
Admission type				**<0** **.** **001**
General ward	45 (54.9%)	43 (63.2%)	2 (14.3%)	
High care	18 (22.0%)	17 (25.0%)	1 (7.14%)	
ICU	19 (23.2%)	8 (11.8%)	11 (78.6%)	
Fever	27 (32.9%)	19 (27.9%)	8 (57.1%)	0.058
Cough	21 (25.6%)	17 (25.0%)	4 (28.6%)	0.75
Respiratory distress	29 (35.4%)	19 (27.9%)	10 (71.4%)	0.004
Hepatomegaly	19 (23.2%)	13 (19.1%)	6 (42.9%)	0.08
Seizures	12 (14.6%)	7 (10.3%)	5 (35.7%)	0.028
Hypotonia/floppiness	10 (12.2%)	3 (4.41%)	7 (50.0%)	<0.001
Hypotension	10 (12.2%)	5 (7.35%)	5 (35.7%)	0.011
Any comorbidity				1
None	32 (39.0%)	27 (39.7%)	5 (35.7%)	
Yes	50 (61.0%)	41 (60.3%)	9 (64.3%)	
Comorbidity				0.75
None	32 (39.0%)	27 (39.7%)	5 (35.7%)	
One	33 (40.2%)	28 (41.2%)	5 (35.7%)	
Two or more	17 (20.7%)	13 (19.1%)	4 (28.6%)	
Comorbidity type				
Asthma	2 (2.44%)	2 (2.94%)	0 (0.00%)	1
CurrentTB	1 (1.22%)	0 (0.00%)	1 (7.14%)	0.17
Past-TB	1 (1.22%)	1 (1.47%)	0 (0.00%)	1
Other CLD	2 (2.44%)	2 (2.94%)	0 (0.00%)	1
Epilepsy	6 (7.32%)	5 (7.35%)	1 (7.14%)	1
Other Chronic neurological disorder	11 (13.4%)	9 (13.2%)	2 (14.3%)	1
Type 1 diabetes	1 (1.22%)	1 (1.47%)	0 (0.00%)	1
CKD	4 (4.94%)	2 (2.99%)	2 (14.3%)	0.14
CVD	10 (12.2%)	8 (11.8%)	2 (14.3%)	0.85
Hypertension	18 (22.0%)	13 (19.1%)	5 (35.7%)	0.18
Malignant neoplasm	16 (19.5%)	15 (22.1%)	1 (7.14%)	0.28
Hematologic disorder	1 (1.22%)	0 (0.00%)	1 (7.14%)	0,17
Chronic liver disease	2 (2.47%)	2 (2.99%)	0 (0.00%)	1
HIV-exposed	18 (22.0%)	12 (17.6%)	6 (42.9%)	0.07
Confirmed HIV-infection				0.43
Negative	79 (96.3%)	66 (97.1%)	13 (92.9%)	
Positive	3 (3.66%)	2 (2.94%)	1 (7.14%)	
Prematurity (<37 weeks)	7 (8.54%)	6 (8.82%)	1 (7.14%)	0.25
Malnutrition	10 (12.3%)	9 (13.2%)	1 (7.69%)	1
MIS-C-physicians impression	7 (8.75%)	5 (7.46%)	2 (15.4%)	0.32
TSS physicians impression	10 (12.3%)	6 (8.82%)	4 (30.8%)	0.05

TB, tuberculosis; CLD, chronic lung disease; CVD, cardiovascular disease; MIS-C, multisystem inflammatory syndrome associated to covid-19; TSS, toxic septic syndrome; MR, mitral regurgitation; TR, tricuspid regurgitation; PHT, pulmonary hypertension.

Meaningful findings are highlighted in bold.

### Biological and microbiological characteristics, as well as treatment received, of PCR-confirmed SARS-CoV-2 children by survival status

**Figure 1:**
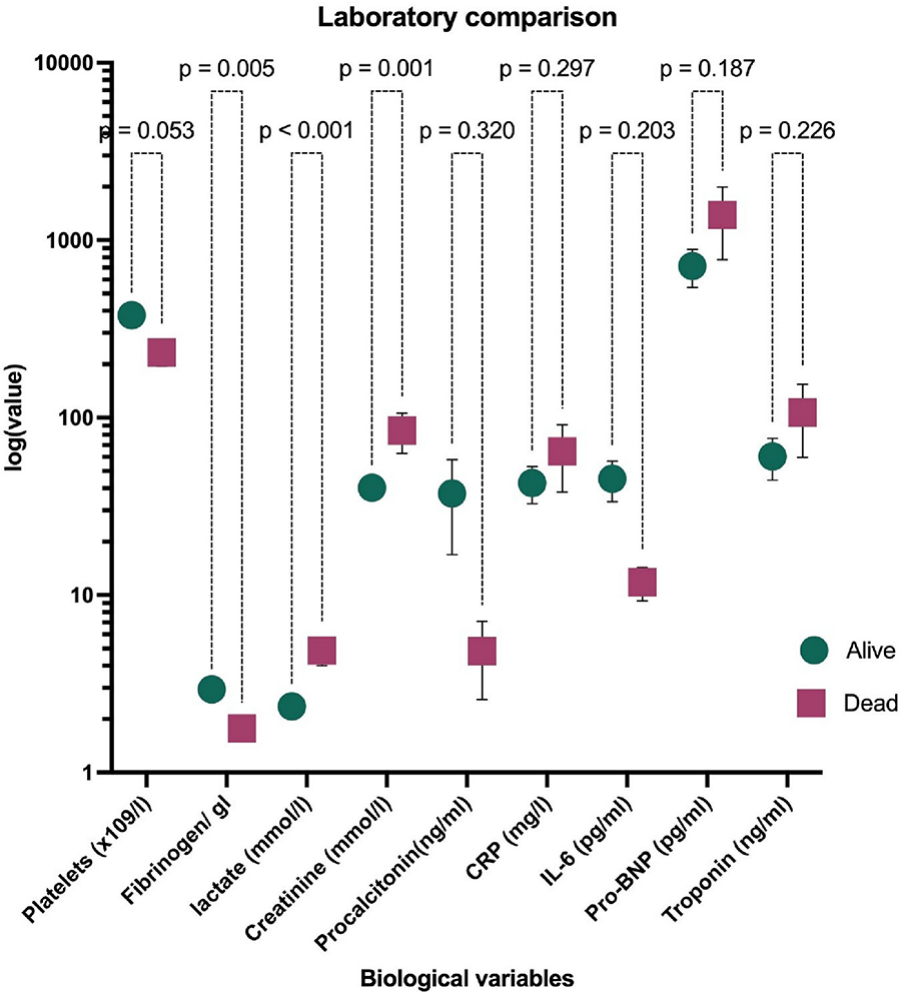
Laboratory findings of PCR-confirmed SARS-CoV-2 children hospitalised at a quaternary health care facility in KwaZulu-Natal South Africa during the first and second waves of COVID-19 pandemic and compared by survival status. Data are represented as median and IQR. The ordinate axis is at log 10 scale. Data at linear scale are in Supplementary Table S1. CRP, C reactive protein; IL-6, interleukin 6; Pro-BNP, pro B-type natriuretic protein.

At admission, children in this cohort who were at risk of death exhibited a significant impairment in coagulation, with low mean levels of fibrinogen (1.77 ± 0.87) and a decrease in platelet count (378 ± 244 vs. 233 ± 143 × 109/l, *p* = 0.006) (Figure 1 and Supplementary Table S1). Additionally, children who died had significantly higher mean levels of lactate (2.36 ± 1.53 vs. 4.87 ± 3.27, *p* = 0.053), and cardiac enzymes tended to be elevated in those at risk of death (Troponin 70.6 vs. 60.4 and Pro-BNP 714 vs. 1,387).

The presence of a culture-proven infection in children was associated with a significant risk of death regardless of the type of pathogen or the source of the infection. In our cohort, 28/82 (34%) cases had confirmed health care associated infections, and 9 of those children died (Table 2). Compared to children who survived, those who died had a significant increase in the use of corticosteroids, antibiotics, oxygen therapy (35.3%), inotropic support (13.4%), blood transfusion (17%), and any type of advanced ventilatory support (27%). This difference in intensive care management was statistically significant.

**Table 2:** Imaging and microbiology findings and type of treatment received of PCR-confirmed SARS-CoV-2 children hospitalised at a quaternary health care facility in KwaZulu-Natal South Africa during the first and second waves of COVID-19 pandemic and classified by survival status.

Variables	All	Alive	Dead	*p*-values
	*N* = 82	*N* = 68	*N* = 14	
Infiltrate pneumonic change on CXR	28 (57.1%)	18 (48.6%)	10 (83.3%)	0.08
Echo findings				0.32
Normal	2 (8.70%)	2 (13.3%)	0 (0.00%)	
MR_TR	9 (39.1%)	7 (46.7%)	2 (25.0%)	
PHT	4 (17.4%)	1 (6.67%)	3 (37.5%)	
Other	8 (34.8%)	5 (33.3%)	3 (37.5%)	
Echo type MISC	15 (65.2%)	8 (53.3%)	7 (87.5%)	0.18
Pathogens identification				0.005
Not done	24 (29.3%)	24 (35.3%)	0 (0.00%)	
Negative	30 (36.6%)	25 (36.8%)	5 (35.7%)	
Positive	28 (34.1%)	19 (27.9%)	9 (64.3%)	
Pathogens identified types				0.85
Gram negative	13 (50.0%)	8 (44.4%)	5 (62.5%)	
Gram positive	11 (42.3%)	8 (44.4%)	3 (37.5%)	
Mixed	1 (3.85%)	1 (5.56%)	0 (0.00%)	
Fungal	1 (3.85%)	1 (5.56%)	0 (0.00%)	
HAI	17 (60.7%)	10 (52.6%)	7 (77.8%)	0.25
Systemic corticosteroids	17 (20.7%)	10 (14.7%)	7 (50.0%)	0.007
IV immunoglobulin	7 (8.54%)	4 (5.88%)	3 (21.4%)	0.09
Antibiotic	44 (53.7%)	34 (50.0%)	10 (71.4%)	0.24
Antibiotic type				0.009
Aminoglycoside	1 (2.27%)	0 (0.00%)	1 (10.0%)	
Betalactam	27 (61.4%)	24 (70.6%)	3 (30.0%)	
Carbapenem	5 (11.4%)	1 (2.94%)	4 (40.0%)	
Glycopeptide	7 (15.9%)	5 (14.7%)	2 (20.0%)	
Macrolide	1 (2.27%)	1 (2.94%)	0 (0.00%)	
Polymyxin	1 (2.27%)	1 (2.94%)	0 (0.00%)	
Sulfamide	2 (4.55%)	2 (5.88%)	0 (0.00%)	
Needs for oxygen	29 (35.8%)	16 (23.9%)	13 (92.9%)	<0.001
Oxygen flow				0.008
1–5 L/min	14 (50.0%)	10 (66.7%)	4 (30.8%)	
6–10 L/min	1 (3.57%)	1 (6.67%)	0 (0.00%)	
11–15 L/min	2 (7.14%)	2 (13.3%)	0 (0.00%)	
>15 L/min	11 (39.3%)	2 (13.3%)	9 (69.2%)	
More than 5 L/min Oxygen				0.13
<5 L/min	14 (50.0%)	10 (66.7%)	4 (30.8%)	
≥5 L/min	14 (50.0%)	5 (33.3%)	9 (69.2%)	
Any invasive ventilation	17 (20.7%)	8 (11.8%)	9 (64.3%)	<0.001
Inotropes vasopressors	11 (13.4%)	3 (4.41%)	8 (57.1%)	<0.001
HFOV	5 (6.17%)	1 (1.49%)	4 (28.6%)	0.003
Blood transfusion	14 (17.1%)	7 (10.3%)	7 (50.0%)	0.002
Days in ICU (mean/SD)	10.8 (14.8)	10.8 (17.8)	10.8 (7.73)	1.00
Days in ICU (median/IQR	7.5 (0.5–13.5)	2 (0–12)	12 (3–14)	0.12
Days Hospitalisation (median/IQR)	7.5 (2–16)	6.5 (2–16.5)	13 (7–16)	0.28

WBC, white blood cells; CRP, C-reactive protein; BNP, B-natriuretic peptide; HFOV, high frequency oscillation ventilation; ICU, intensive care unit; HAI, health care associated infection; MISC, multisystem inflammatory syndrome associated to covid-19; IQR, interquartile range; SD, standard deviation; MR-TR, mitral regurgitation-tricuspid regurgitation; PHT, pulmonary hypertension; CXR, chest x-ray.

### Predictors associated with need for intensive care in children with PCR-confirmed SARS-CoV-2

After stratification by age group and gender, children admitted to IALCH were 5.4 times more likely to be at risk of need for intensive care. This difference was statistically significant (*p* = 0.02) (Table 3). In a model that considered age, gender, and the presence of more than 1 comorbidity, every one-year increase in age reduced the risk of need for intensive care by 14%, with estimates ranging from 7% to 20%, *p* < 0.001. Children with more than one comorbidity had a 2.47-fold increased risk of need for intensive care (aPR:2.47, 95%CI: 1.32–4.61, *p* = 004). After adjusting comorbidities by age and gender, the impact of HIV exposure lost its statistical significance and was diluted. In this model, chronic kidney diseases (CKD) retained its statistical significance as a variable, as children diagnosed with CKD exhibited a 4.68-fold (95% CI: 1.30–16.86, *p* = 0.02) higher likelihood of requiring critical care. Further, an increase in creatinine and blood urea nitrogen (BUN) levels by 1 unit was significantly associated with a 10% (with estimates ranging from 1% to 15%, *p* < 0.001) and a 20% (with estimates ranging from 3% to 32%, *p* = 0.02) increase in the risk of need for intensive care, respectively, independently. Regardless of age, gender, and comorbidities, children presenting with seizures were at 2.39-fold increased risk of need for intensive care, with estimates ranging from 1.56 to 3.68, *p* < 0.001.

**Table 3:** Predictors associated with need for critical care among PCR-confirmed SARS-CoV-2 children hospitalised at a quaternary health care facility in KwaZulu-Natal South Africa during the first and second waves of COVID-19 pandemic.

	aPR	Lower limit	Upper limit	*p*-value
Model 1: Effect of age and gender on the need for critical care				
Age-group				
<1 year	3.02	1.05	8.66	0.04
1–4 years	2.30	0.69	7.63	0.18
>4 years	1.17	0.28	4.85	0.83
Gender	0.71	0.39	1.29	0.26
Model 2^a^: Effect of comorbidity on the need for critical care				
Age (continuous)	0.86	0.80	0.93	*p* < 0.001
More than 1 comorbidities				
No	0.92	0.44	1.91	0.83
Yes	2.47	1.32	4.61	0.004
Model 3^a^: Effect of comorbidities’ type on the need for critical care				
CVD/yes	1.40	0.72	2.73	0.32
CKD/yes	4.68	1.30	16.83	0.018
Neoplasm/yes	0.98	0.31	3.06	0.98
HIV-infection/yes	1.44	0.78	2.67	0.25
Model 4^a^: Effect seizure at presentation on the need for critical care				
Seizures at admission/yes	2.39	1.56	3.68	<0.001
Model 5^a^: Effect of biological findings on the need for critical care				
Creatinine (mmol/L)	1.01	1.001	1.015	<0.001
BUN (mmol/L)	1.02	1.003	1.032	0.02

Models 2–5 are adjusted for age as continuous variable and for gender as binary variable.

^a^adjusted.

aPR, adjusted prevalence ratios; CVD, cardiovascular disease; CLD, chronic liver diseases; WBC, white blood cell; BUN, blood urea nitrogen; CKD, chronic kidney diseases.

### Predictors associated with in-hospital death in children with PCR-confirmed SARS-CoV-2

The results of our study indicate that several factors are independently associated with in-hospital mortality among children with confirmed COVID-19 infection (Table 4). We found that mortality was significantly predicted by exposure to HIV infection, the need for invasive ventilation and increased in BUN, with adjusted hazard ratios (aHRs) of 6.8 (95%CI: 1.54–31.71, *p* = 0.011), 3.59 (95%CI: 1.01–12.16, *p* = 0.048) and 1.06 (95%CI: 1.01–1.11, *p* = 0.017), respectively. Nonetheless, after adjusting for age, we also observed that male children and those primarily admitted from home to the quaternary hospital were less likely to die from COVID-19, with aHRs of 0.12 (95%CI: 0.01–0.95, *p* = 0.05) and 0.23 (95%CI: 0.1–0.86, *p* = 0.029), respectively. Additionally, decreased platelet, increased creatinine levels, history of CKD and respiratory distress at admission were also associated with mortality at a borderline statistical or clinical significance.

**Table 4:** Predictors associated with in-hospital mortality among PCR-confirmed SARS-CoV-2 children hospitalised at a quaternary health care facility in KwaZulu-Natal South Africa during the first and second waves of COVID-19 pandemic and classified by survival status.

Variables	cHR [95%CI]	*p* value	aHR [95%CI]	*p* value
Model 1: Effect of age, gender and HCF level facility on in-hospital death				
Age group				
<1 year	1.29 [0.34–4.94]	0.71	2.21 [0.54–9.12]	0.27
1–4 years	0.43 [0.04–4.11]	0.46	0.43 [0.04–4.72]	0.5
5–9 years	0.73 [0.12–4.48]	0.74	1.28 [0.21–8.1]	0.79
10–12 years	Ref.			
Gender/male	0.13 [0.02–1.03]	0.054	0.12 [0.01–0.95]	0.05
Primary admission at QHCF/yes	0.29 [0.09–0.91]	0.03	0.23 [0.1–0.86]	0.029
Model 2: Effect of clinical presentation on in-hospital death				
Respiratory distress/yes	3.81 [1.19–12.19]	0.02	4.21 [0.86–20.68]	0.08
TSS-Physician/yes	1.98 [0.60–6.51]	0.26	2.21 [0.49–9.87]	0.29
MIS-C/yes	0.93 [0.20–4.24]	0.92	0.32 [0.06–1.68]	0.18
Splenomegaly/yes	0.66 [0.09–5.22]	0.70	1.96 [0.17–22.3]	0.59
Model 3: Effect of HIV status or HIV exposure on in-hospital death				
Child HIV(+)/yes	13.43 [1.22–148.21]	0.03	4.3 [0.37–51.01]	0.24
HIV infection exposed/yes	4.99 [1.50–16.63]	0.009	6.8 [1.54–31.71]	0.01
Model 4: Effect of comorbidities’ type on in-hospital death				
CVD/yes	0.62 [0.20–1.91]	0.41	0.74 [0.14–3.90]	0.72
CKD/yes	3.55 [0.76–16.53]	0.11	8.15 [0.85–78.46]	0.07
HIV infection exposed/yes	4.99 [1.50–16.63]	0.009	7.69 [1.42–41.56]	0.018
Malnutrition/yes	1.03 [0.13–8.08]	0.98	0.57 [0.07–4.97]	0.61
Chronic neurologic disorders/yes	0.58 [0.13–2.59]	0.47	1.20 [0.21–7.00]	0.84
Model 5: Effect of laboratory values on in-hospital death				
Platelets (×10^9^/L)	0.996 [0.993–0.999]	0.027	0.99 [0.98–0.999]	0.04
Creatinine (mmol/L)	1.01 [0.99–1.01]	0.06	1.01 [0.99–1.02]	0.07
BUN (mmol/L)	1.05 [1.01–1.11]	0.03	1.06 [1.01–1.11]	0.017
Model 6: Effect of treatment received on in-hospital death				
Any invasive ventilation/yes	4.21 [1.40–12.71]	0.01	3.59 [1.01–12.75]	0.048
Steroids/yes	1.88 [0.41–8.59]	0.42	0.9 [0.18–4.45]	0.89
Antiviral/yes	0.84 [0.19–3.80]	0.82	0.78 [0.19–3.83]	0.76

Models 2–6 are adjusted for age as continuous variable and for gender as binary variable. HIV infection exposed = child born from known mother living with HIV-infection.

cHR, crude hazard ratio; aHR, adjusted hazard ratio; HCF, health care facility; QHCF, quaternary HCF; CVD, cardio-vascular diseases; CKD, chronic kidney disease; BUN, blood urea nitrogen; TSS, toxic shoch syndrome; MIS-C, multisystem inflammatory syndrome in children.

## Discussion

Our study presents a summary of the results obtained from a nested study conducted within a large cohort in various African countries. The study focused on children who were hospitalized with COVID-19 infection between March 2020 and December 2021 in KwaZulu-Natal South Africa. We analysed the 10-day outcome of these children, with a particular focus on risk of ICU admission, oxygen supplementation and in-hospital mortality. The median age of the study population was 4.1 years, and there was a slight female predominance (1.7:1). Our findings showed that 23.1% of the children required ICU admission, 35.3% needed oxygen supplementation, and among these children, 27% received some form of ventilation. The overall mortality rate was significantly high at 17% which highlights the severity of COVID-19 in children with underlying medical conditions. Furthermore, the findings indicated that children with pre-existing medical conditions were at higher risk of requiring critical care and death, with exposure to HIV-infection and younger age being among significant predictors.

Our cohort reported a higher mortality rate compared to previous multicentre studies (17–22), with similar factors associated with ICU admission and death, except for the particularity of HIV exposure and referral (vs. primary) admission to a quaternary health care facility. As such, the higher mortality rate observed in our cohort could be attributed to delays in referral to appropriate facilities and seeking medical help. A study conducted in South Africa reported a relatively high mean ambulance transport time of 4.9 h from the district hospital to the regional hospital (23). Additionally delays in seeking care for children with pneumonia have been linked to factors such as distance to health facilities, lack of skills to recognize emergencies, lack of confidence in the health system, and self-medication practices (24). The inadequate healthcare delivery, including the lack of appropriate equipment including lack of ICU beds and staffing in the public sector, could also contribute to the high mortality rates during the COVID-19 pandemic (25). These findings constitute a call for reducing health inequity by expending universal health coverage to achieve a resilient health system in regions with variables resources and expansion of intensive care facilities closer to where patients live.

Furthermore, studies investigating the association between HIV infection and COVID-19 outcomes have reported mixed results. Some studies have found no significant association, while others have found that people living with HIV (PLWH) may be at an increased risk of severe COVID-19 disease. Recent studies conducted in South Africa and the United States have shown that PLWH, particularly those with comorbidities, Black or Hispanic ethnicity, and HIV-infected children, are at a greater risk of hospitalization and death due to COVID-19. Despite these findings, further research is needed to fully understand the relationship between HIV infection and COVID-19 outcomes (26–28). Although we did not observe a direct association between confirmed HIV infection and increased mortality (due to limited number in this category, *n* = 3), we did find that exposure to HIV-infection played an independent role in COVID-19 outcomes for children. This novel finding agrees with previous studies suggesting that HIV exposure may increase the risk of infectious diseases in children, even if they are uninfected with HIV. For example, a meta-analysis pooling data from 35 studies and aimed to compare the incidence of diarrhoea and pneumonia in HIV-exposed uninfected (HEU) children and HIV-unexposed uninfected (HUU) children (29). The study found that HEU children had a higher risk of diarrhoea and pneumonia than HUU children. These results suggest that HEU children may benefit from targeted interventions to reduce their risk of these infections. Another hypothesis generated is that this exposure is a proxy for delayed HIV diagnosis and healthcare access, which may contribute to poorer COVID-19 outcomes. Additionally, healthcare-associated infections were observed in 20% of the assessed children during their hospitalization, which could have potentially influenced their outcomes, although didn’t reach statistical significance, but still having clinical significance.

In our study, severe disease in children was characterized by a combination of independent clinical and biological indicators including respiratory failure requiring ventilation, myocarditis indicated by elevated cardiac enzymes, shock manifested as hyperlactaemia and hypotension, acute kidney failure indicated by elevated creatinine and urea, and signs of neurological involvement such as seizures and hypotonia. These findings are consistent with those reported in previous studies of children from various populations (30–33). The fact that 27% of children who received oxygen supplementation required some form of ventilation indicates the severity of the disease in these patients. This highlights the importance of ensuring that adequate medical resources, such as ventilators, are available in healthcare facilities caring for children with COVID-19 in the African region. In the studied population, interim ventilation facilities are available outside an ICU setting, which may reflect the quality of care that is provided to these children outside of a formal intensive care unit. Another significant finding was the association between age and COVID-19 outcomes in children. The study found that younger children (less than 1 year old) were at a higher risk of requiring critical care. This finding is consistent with other studies that have found that children under 1 year of age are at a higher risk of severe COVID-19 outcomes (34, 35). The reported median age of 4.1 years is consistent with early studies conducted in Europe (17–20) as well as studies in Africa and Latin America, while studies in China and the United States reported higher median ages (22, 36).

Furthermore, the association between comorbidities and critical care need and death is consistent with previous studies that have shown comorbidities as a risk factor for severe COVID-19 in children (10, 30). While comorbidities such as CKD and signs of kidney failure identified as independent risk factors for severe COVID-19 outcomes in children, this was not the case for CVD and prematurity. This may be due to the lack of routine cardiological assessment during the pandemic's early stages in our setting, the important use of systemic corticoids and the limited numbers in our sample. Similarly, records of prematurity may have been missing in patient files. However, the study's findings regarding the association between male gender and a lower risk of death are somewhat conflicting, as some studies have reported a higher incidence of severe disease in boys (37), while others have found no significant difference (11).

Our findings will be interpreted in light of some limitations. Firstly, it was conducted in a single and quaternary health care facility and from a high HIV burden setting, which may introduce the risk of selection bias and limit the generalizability to other settings. Secondly, the sample size was relatively small, and the study may have been underpowered to detect some significant differences. Finally, due to the retrospective nature of the study design, there may have been some missing data or unmeasured confounding factors that could have influenced the results. This might include the lack of adjustment for circulating variant of concern among hospitalised children which could have influenced the severity of illness. Notably, our study was one of the first to investigate the impact of HIV exposure in a high-prevalence setting, demonstrating its significant association with mortality. Nationwide prospective cohort considering variant of concerns identification and more diverse populations are needed to better inform policy on the observed findings.

## Conclusion

Our findings highlight the importance of providing special attention to children with underlying medical conditions in preventing and managing COVID-19 as they are at increased risk of dying with COVID-19. As such, when allocating intensive care resources during COVID-19 surges, age, and comorbidity, particularly for infants under one year old or those exposure to HIV infection, should be considered. Further, access to tertiary/quaternary facilities to overcome healthcare delivery challenges in Africa, such as delayed referral, insufficient equipment, lack of sufficient ICU beds and staffing shortage at different levels of care, which may contribute to high mortality rates.

## Data Availability

The raw data supporting the conclusions of this article will be made available by the authors, without undue reservation.
