# Supplementary material for: Predictors associated with critical care need and in-hospital mortality among children with laboratory-confirmed COVID-19 infection in a high HIV infection burden region
Source: Front Pediatr. 2023 Sep 7;11:1252886. doi: 10.3389/fped.2023.1252886 (PMC10512976; doi:10.3389/fped.2023.1252886)
Supplement: Supplementary file 1 [file Table1.docx]

**Supplemental Table 1. Laboratory characteristics of included participants**

| **Variables** | **All** | **Alive** | **Dead** |
| --- | --- | --- | --- |
|  | n=82 | n=68 | n=14 |
| **Haemoglobin (g/l)** | 10.9 (2.52) | 11.1 (2.55) | 10.1 (2.29) |
| **Total WBC count (x10^9^/l)** | 10.8 (8.63) | 10.8 (8.85) | 10.8 (7.92) |
| **Platelets (x10^9^/l)** | 350 (234) | 378 (244) | 233 (143) |
| **Fibrinogen/ gl** | 2.68 (1.32) | 2.94 (1.33) | 1.77 (0.87) |
| **lactate (mmol/l)** | 2.95 (2.29) | 2.36 (1.53) | 4.87 (3.27) |
| **Creatinine (mmol/l)** | 48.8 (45.9) | 40.3 (27.3) | 84.6 (81.4) |
| **Procalcitonin(ng/ml)** | 29.2 (147) | 37.4 (169) | 4.84 (8.47) |
| **CRP (mg/l)** | 47.9 (86.6) | 42.8 (83.1) | 64.5 (99.1) |
| **D-dimer (mg/l)** | 3.30 (5.01) | 3.30 (4.69) | 3.32 (6.32) |
| **IL-6 (pg/ml)** | 38.2 (86.3) | 45.2 (96.4) | 11.8 (9.45) |
| **Glucose (mmol/l)** | 5.80 (4.23) | 5.53 (3.80) | 6.88 (5.70) |
| **Pro-BNP (pg/ml)** | 882 (1616) | 714 (1422) | 1387 (2280) |
| **Troponin (ng/ml)** | 70.6 (140) | 60.4 (132) | 107 (177) |
| **BUN** **(mmol/l)** | 5.3(5.84) | 4.5(2.7) | 8.7(11.9) |
